# A survival comparison of gastric mucin-producing adenocarcinoma to conventional adenocarcinoma: a SEER database analysis

**DOI:** 10.1186/s12885-021-08835-z

**Published:** 2021-10-23

**Authors:** Qiang Wang, Junbin Zhong, Qing Huang, Zhuanpeng Chen, Jianchang Wei, Fang Wei, Jie Cao

**Affiliations:** 1 The First Affiliated Hospital, Jinan University, Guangzhou, Guangdong China; 2 Department of General Surgery, Guangzhou Digestive Disease Center, Guangzhou First People’s Hospital, School of Medicine, South China University of Technology, Guangzhou, Guangdong China

**Keywords:** Gastric mucin-producing adenocarcinoma, Survival, SEER database

## Abstract

**Background:**

Compared to conventional adenocarcinoma (CA), mucin-producing adenocarcinoma (MPA) is an uncommon histological subtype and is usually separated from other histological types and has been evaluated separately. The objective was to compare the clinicopathological characteristics and survivals of MPA with CA.

**Methods:**

We retrospectively analyzed 1515 MPA patients in SEER database. Log-rank tests and KM survival curves were applied to determine the differences in overall survival (OS) and cancer specific survival (CSS) time.

**Results:**

No significant differences were noted in OS and CSS time. The MPA patients who were treated with surgery and chemotherapy exhibited longer OS and CSS time periods than those without treatment. MPA patients treated with radiotherapy exhibited similar OS and CSS time with those without radiotherapy. MPA was not a prognostic factor of survival.

**Conclusions:**

MPA was a rare histological type of gastric cancer. Patients with MPA exhibited similar prognosis with those with CA. Surgery and chemotherapy were effective treatments for patients with MPA.

## Background

Gastric cancer (GC) is an important digestive cancer encountered worldwide that ranks the fifth most frequently diagnosed cancer and the third leading cause of cancer death [1].

GC is histologically divided into two categories as follows: differentiated and undifferentiated cancer [2]. Differentiated cancers include well differentiated, moderately differentiated and papillary adenocarcinomas, whereas, undifferentiated tumors include signet ring cell carcinoma, poorly differentiated and mucinous adenocarcinomas [3]. Compared to differentiated cancer, undifferentiated cancer demonstrates aggressive growth, proliferation, invasiveness, metastasis and poor prognosis [3].

Mucin-producing adenocarcinoma (MPA), such as mucinous adenocarcinoma and signet ring cell carcinoma, have the characteristics of producing mucin by the presence of intracellular or extracellular mucin pool [4]. Mucinous adenocarcinoma is characterized with more than 50% of extracellular mucin, and signet-ring cell carcinoma is characterized with more than 50% cells that contain intracellular mucin. The latter cause a certain movement of the nucleus to one side in order to create the characteristic morphology [5]. Compared to conventional adenocarcinoma (CA), MPA is an uncommon histological subtype and is considerably different in morphology, cell characteristics and protein expression [6]. Therefore, MPA is usually separated from other histological types and has been evaluated separately [3].

The clinicopathological characteristics and the prognosis of gastric MPA patients have been investigated in several studies. However, the results are inconsistent and the prognostic significance of MPA is still unclear. MPA was initially reported to be correlated with unfavorable prognosis than other histological types [7–10]. In contrast to these findings, other studies demonstrated no significant differences in disease prognosis [11–15]. MPA has not been reported as a negative prognostic factor [16–18]. It is important to state that signet ring cell carcinoma is associated with a favorable prognosis compared with other types of gastric cancer [19–22].

Therefore, further investigations are essential in identifying the clinicopathological characteristics and prognostic value of MPA. In the present study, we retrospectively analyzed 1515 MPA gastric cancer patients in the Surveillance, Epidemiology and End Results (SEER) database in order to compare the clinicopathological characteristics and survival outcomes of MPA with CA.

## Materials & methods

### Patient selection

Clinicopathological data derived from gastric cancer patients were collected from the SEER database (18 Regs Custom Data with additional treatment fields,, 1975–2016 varying) using SEER*Stat 8.3.8 (http://seer.cancer.gov). The patient information in the SEER database is anonymous and ethical consent was not required for using these data [23, 24].

We collected the information of the patients diagnosed with GC from the SEER database during 2004–2015, because we required the TNM stage information from American Joint Committee on Cancer (AJCC) 6th (2004) edition for analysis.

The histological type of GC was defined as follows: code 8140 corresponded to adenocarcinoma and codes 8480 (Mucinous adenocarcinoma), 8481(Mucin-producing adenocarcinoma) and 8490 (Signet ring cell carcinoma) to mucin-producing adenocarcinoma.

The clinicopathological information of patients including age, sex race, tumor differentiation, tumor invasion, node, metastasis (TNM) stage, surgery, chemotherapy, radiation and survival time was collected from the SEER database.

The patients who < 18 years old, without positive histology, with distant metastasis, with multiple primary tumors, with unknown follow up time and information were excluded.

Overall survival (OS) is the survival time from patient’s diagnosis of the disease to death, and cancer specific survival (CSS) is the survival time from patient’s diagnosis of the disease to death specific attributable to the cancer.

### Statistical analysis

The Chi-square test was applied to determine the differences in the clinicopathological characteristics between the MPA and the CA groups. The log-rank test and Kaplan–Meier survival curve were applied to determine the differences in OS and CSS time periods between the MPA and the CA groups. Cox regression models were applied to assess the association between histological types and OS following adjustment for potential confounders, such as age, race, married status, tumor differentiation, clinical stage, surgery, chemotherapy and radiotherapy. The analysis was performed by Empower (R) (X&Y solutions, inc. Boston MA, www.empowerstats.com) and R (http://www.R-project.org).

## Results

### Patient characteristics

**Fig. 1:**
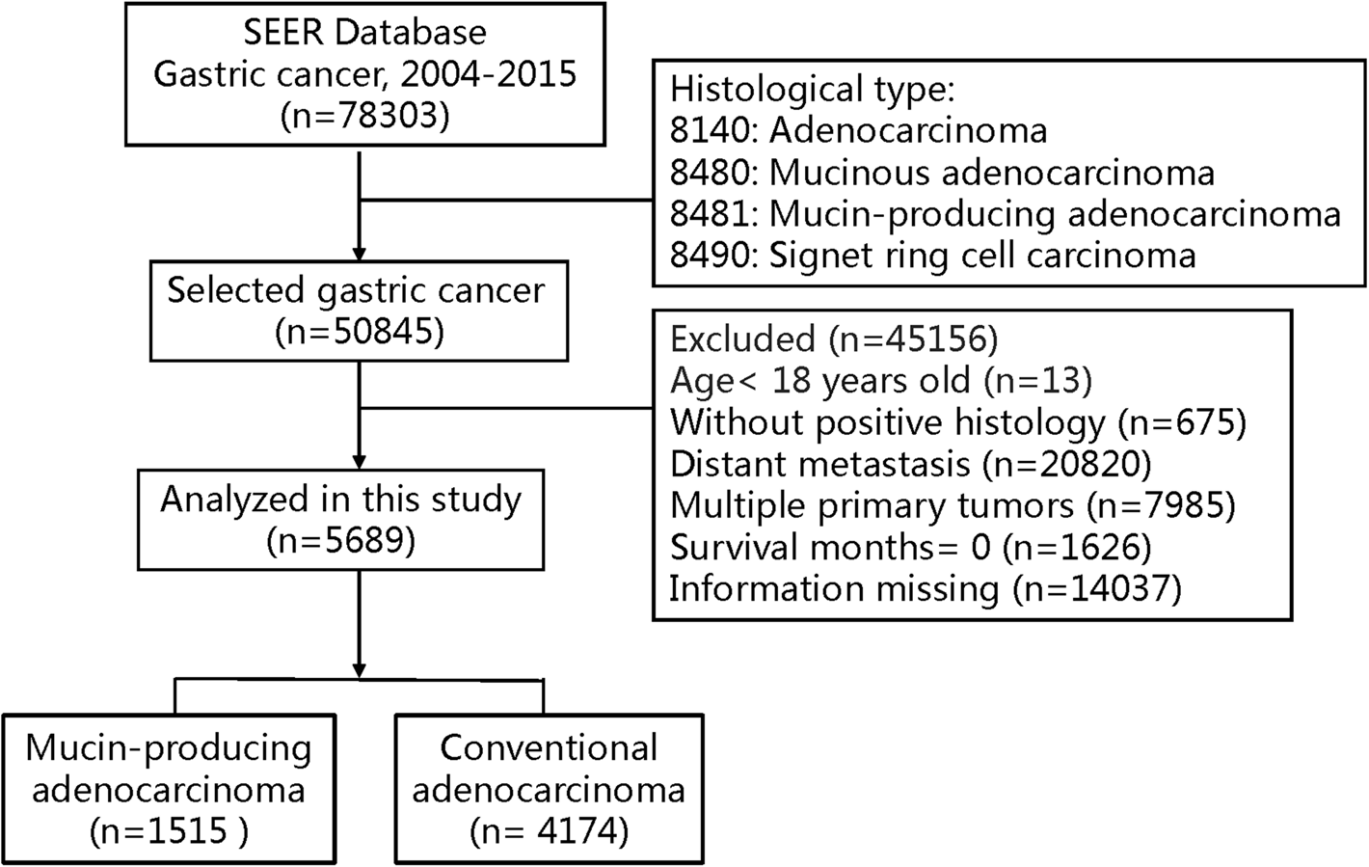
Flowchart of the patients’ selection process

The selection flow of the patients is shown in Fig. 1. A total of 5689 eligible patients were selected from the SEER database during the period 2004–2015, of which 1515 patients (26.6%) presented with MPA and 4174 patients (73.4%) with CA.

The clinicopathological characteristics of the two groups are summarized in Table 1. Overall, age, gender, race, tumor differentiation, clinical stage, T stage, N stage, surgery and chemotherapy were significantly different between MPA and CA, i.e., patients in the MPA group exhibited younger, more females, more black people, more poor/undifferentiated tumors, later clinical stage, more T3, T4, N2 stages, more surgery and chemotherapy than those of the CA group. No difference was noted in radiotherapy.

**Table 1:** Patients demographics and clinicopathological characteristics

Variables	n	MPA n (%)	CA n (%)	P
**Age**				
< 60	1996	692 (45.7)	1304 (31.2)	0.00
≥ 60	3693	823 (54.3)	2870 (68.8)	
**Gender**				
Female	1614	603 (39.8)	1011 (24.2)	0.00
Male	4075	912 (60.2)	3163 (75.8)	
**Race**				
White	4332	1056 (69.7)	3276 (78.5)	0.00
Black	593	192 (12.7)	401 (9.6)	
Others	764	267 (17.6)	497 (11.9)	
**Differentiation**				
Well/ Moderate	1885	121 (8)	1764 (42.3)	0.00
Poor/undifferentiated	3804	1394 (92)	2410 (57.7)	
**Clinical stage**				
I	1764	401 (26.5)	1363 (32.7)	0.00
II	2032	495 (32.7)	1537 (36.8)	
III	1893	619 (40.8)	1274 (30.5)	
**T stage**				
T1	877	193 (12.7)	684 (16.4)	0.00
T2	3089	799 (52.8)	2290 (54.9)	
T3	1564	473 (31.2)	1091 (26.1)	
T4	159	50 (3.3)	109 (2.6)	
**N stage**				
N0	1924	462 (30.5)	1462 (35)	0.00
N1	2861	707 (46.7)	2154 (51.6)	
N2	904	346 (22.8)	558 (13.4)	
**Surgery**				
Yes	4123	1241 (81.9)	2882 (69.1)	0.00
No	1566	274 (18.1)	1292 (30.9)	
**Chemotherapy**				
Yes	5101	1392 (91.9)	3709 (88.9)	0.001
No/ unkown	588	123 (8.1)	465 (11.1)	
**Radiotherapy**				
Yes	5533	1472 (97.2)	4061 (92.3)	0.78
No	156	43 (2.8)	113 (7.7)	

### Survival analysis by histological type

**Fig. 2:**
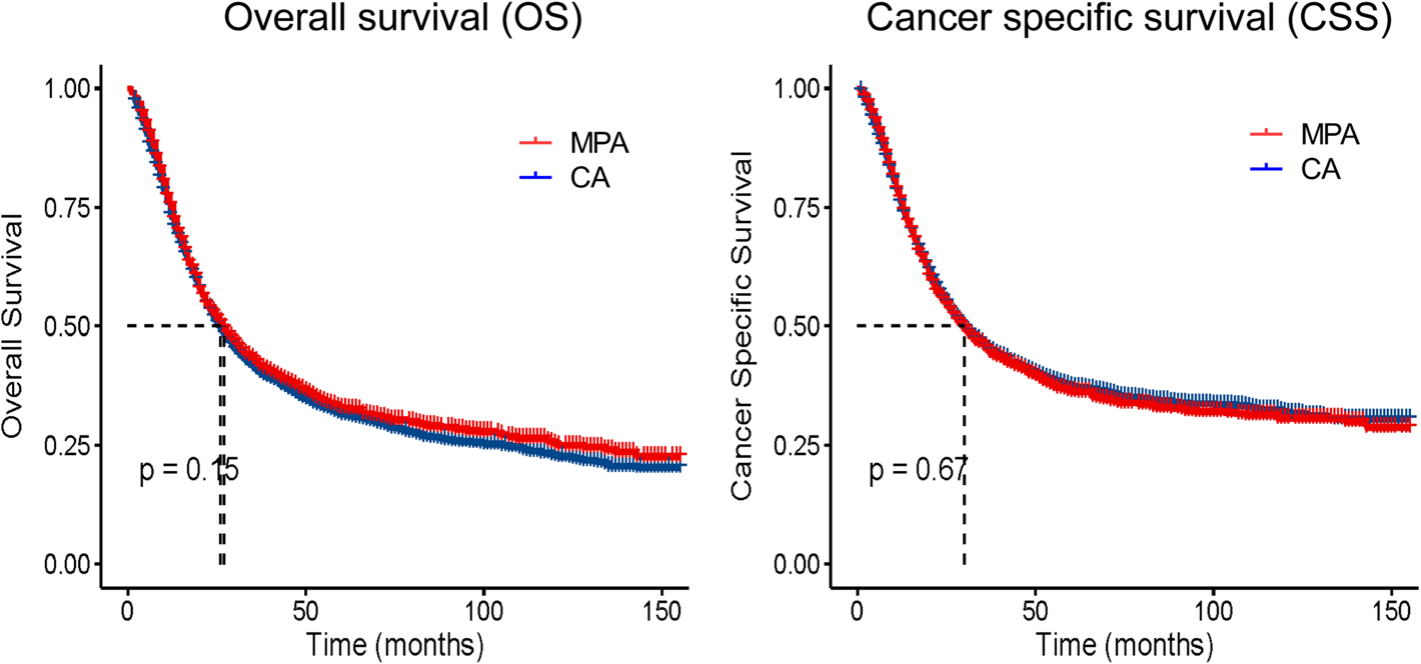
Overall survival (OS) and cancer specific survival (CSS) analysis by different histological types of gastric cancer patients

To investigate whether MPA patients exhibit different survival time with CA patient, we compared the OS and CSS time between the two groups. The OS time in the MPA group was 58.67 ± 1.67 months, whereas that in the CA group was 55.68 ± 1.00 months; however, no significant difference was observed between the two groups (log-rank = 2.07, *P* = 0.15) (Fig. 2). The CSS time in the MPA group was 64.69 ± 1.79 months, whereas that in the CA group was 66 ± 1.13 months; however, no significant difference was observed between the two groups (log-rank = 0.185, *P* = 0.67) (Fig. 2).

**Fig. 3:**
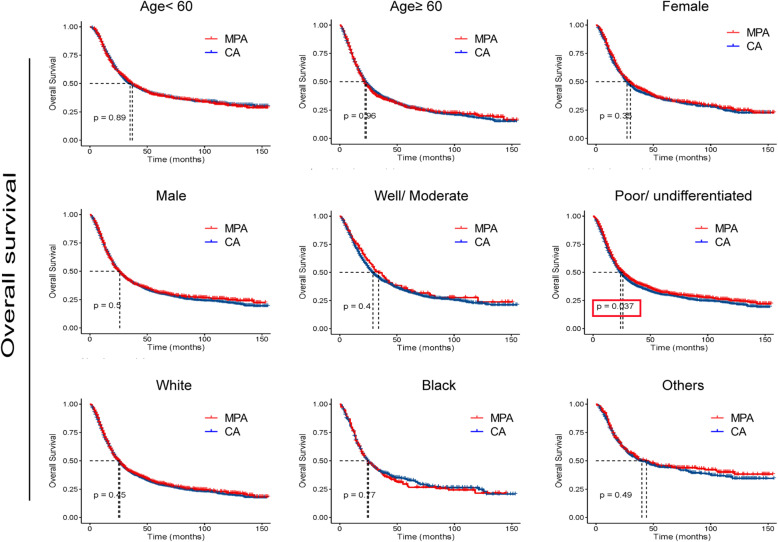
Overall survival (OS) analysis by mucin-producing adenocarcinoma (MPA) and conventional adenocarcinoma (CA) in different clinicopathological groups of gastric cancer patients.

**Fig. 4:**
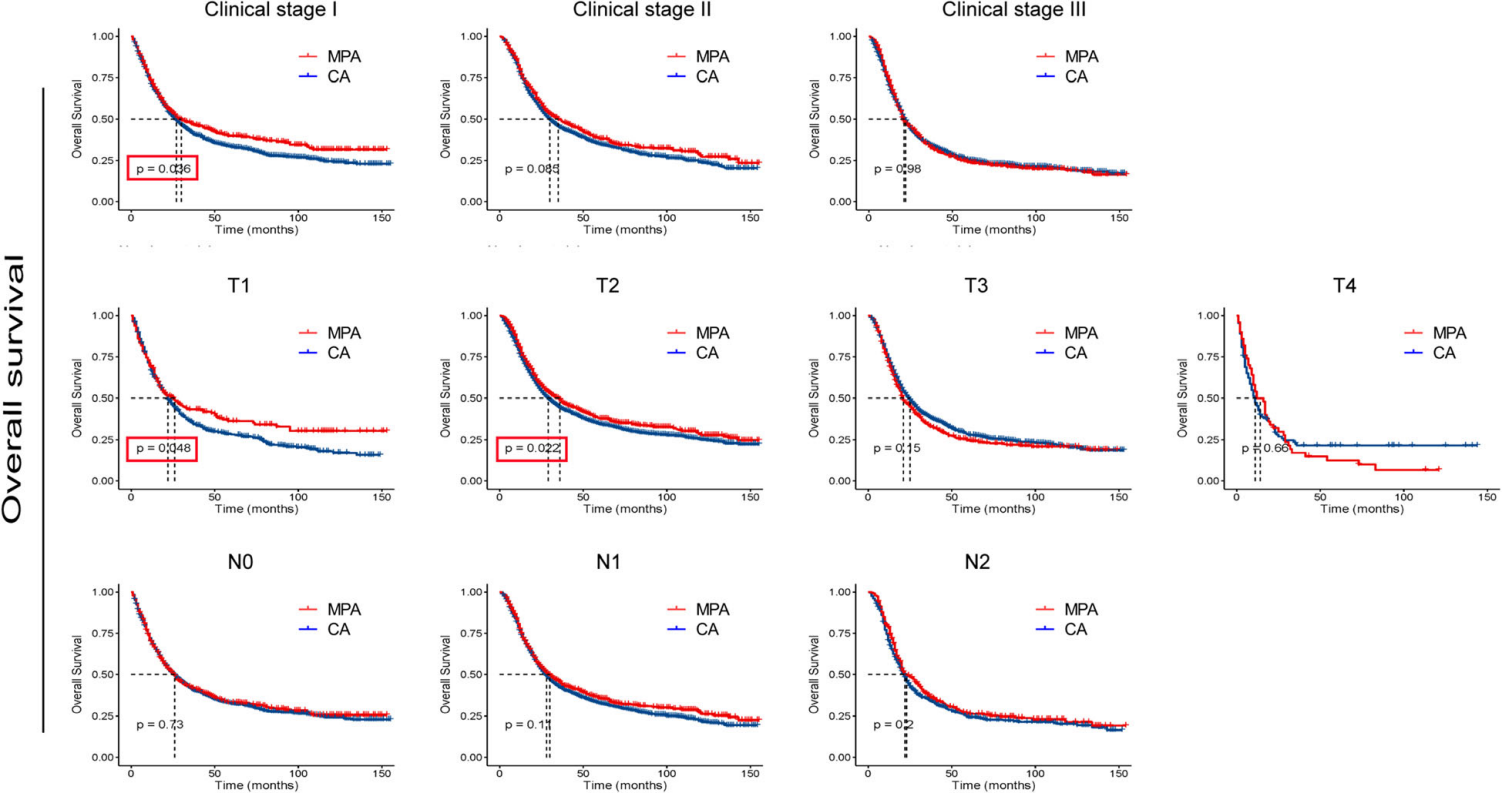
Overall survival (OS) analysis by mucin-producing adenocarcinoma (MPA) and conventional adenocarcinoma (CA) in different clinicopathological stages of gastric cancer patients.

**Fig. 5:**
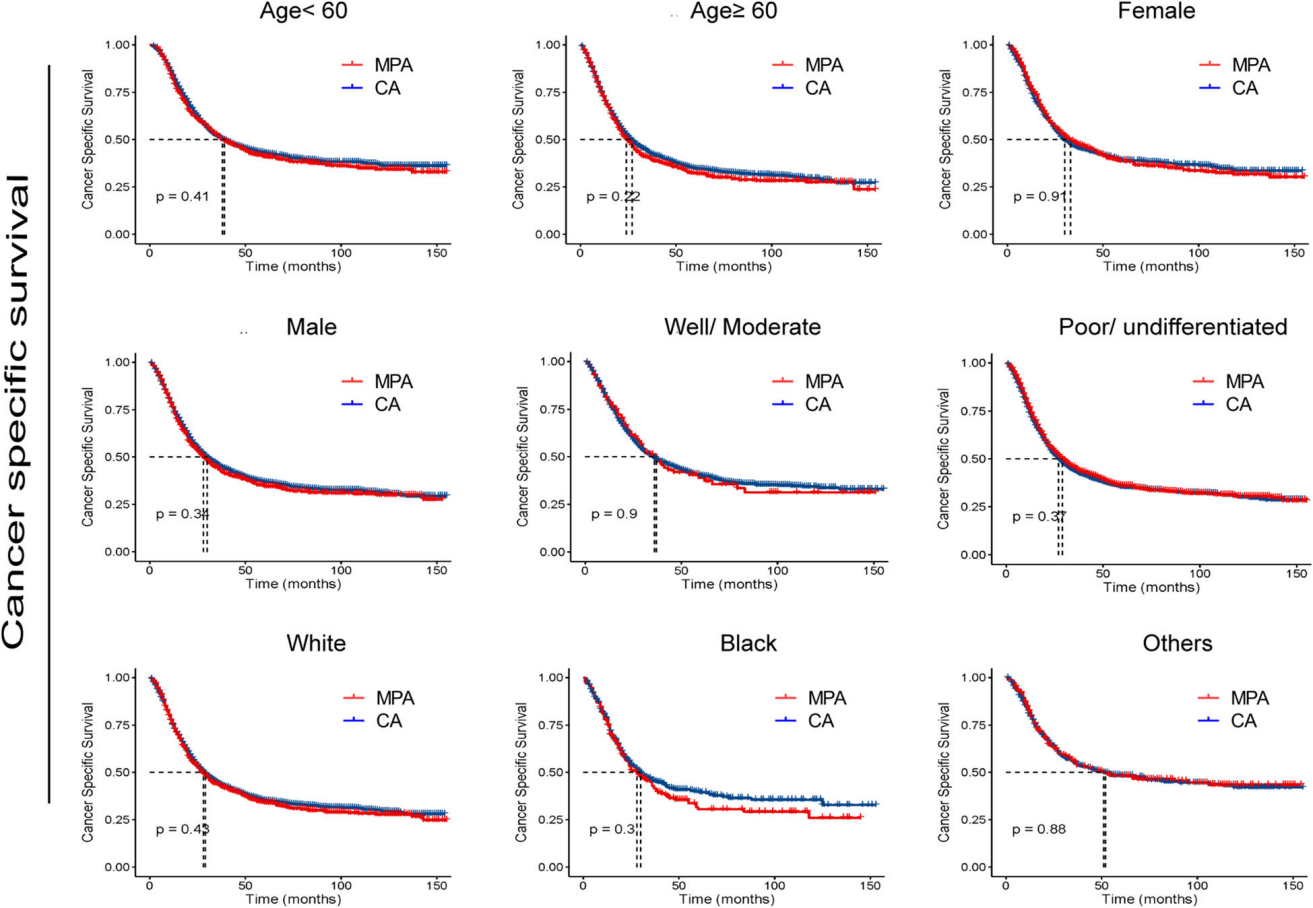
Cancer specific survival (CSS) analysis by mucin-producing adenocarcinoma (MPA) and conventional adenocarcinoma (CA) in different clinicopathological groups of gastric cancer patients.

**Fig. 6:**
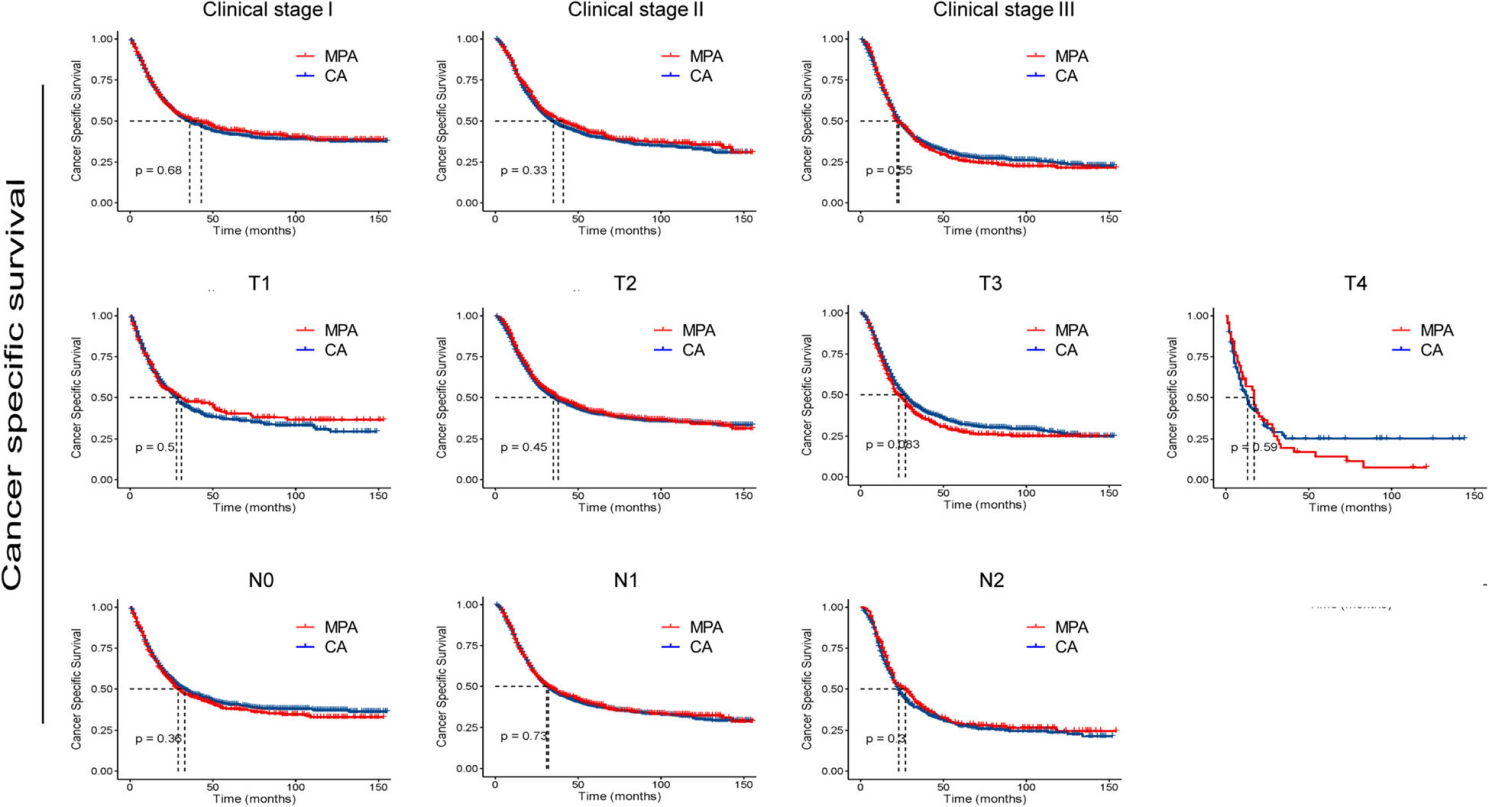
Cancer specific survival (CSS) analysis by mucin-producing adenocarcinoma (MPA) and conventional adenocarcinoma (CA) in different clinicopathological stages of gastric cancer patients.

To further determine whether MPA patients show similar survival time with CA patient in different subgroup, we compared the survival curves. As shown in Figs. 3 and 4, no significant differences were detected in OS times between the two groups with respect to different ages, genders, races and N stages. Interestingly, for patients with poor/ undifferentiated tumor, clinical stage I, T1 and T2, MPA patients had longer OS time than CA patients (*p* < 0.05). As shown in Figs. 5 and 6, no significant differences were detected in CSS times between the two groups with respect to different ages, genders, races tumor differentiation, clinical stages, T stages and N stages (All *p* > 0.05).

**Fig. 7:**
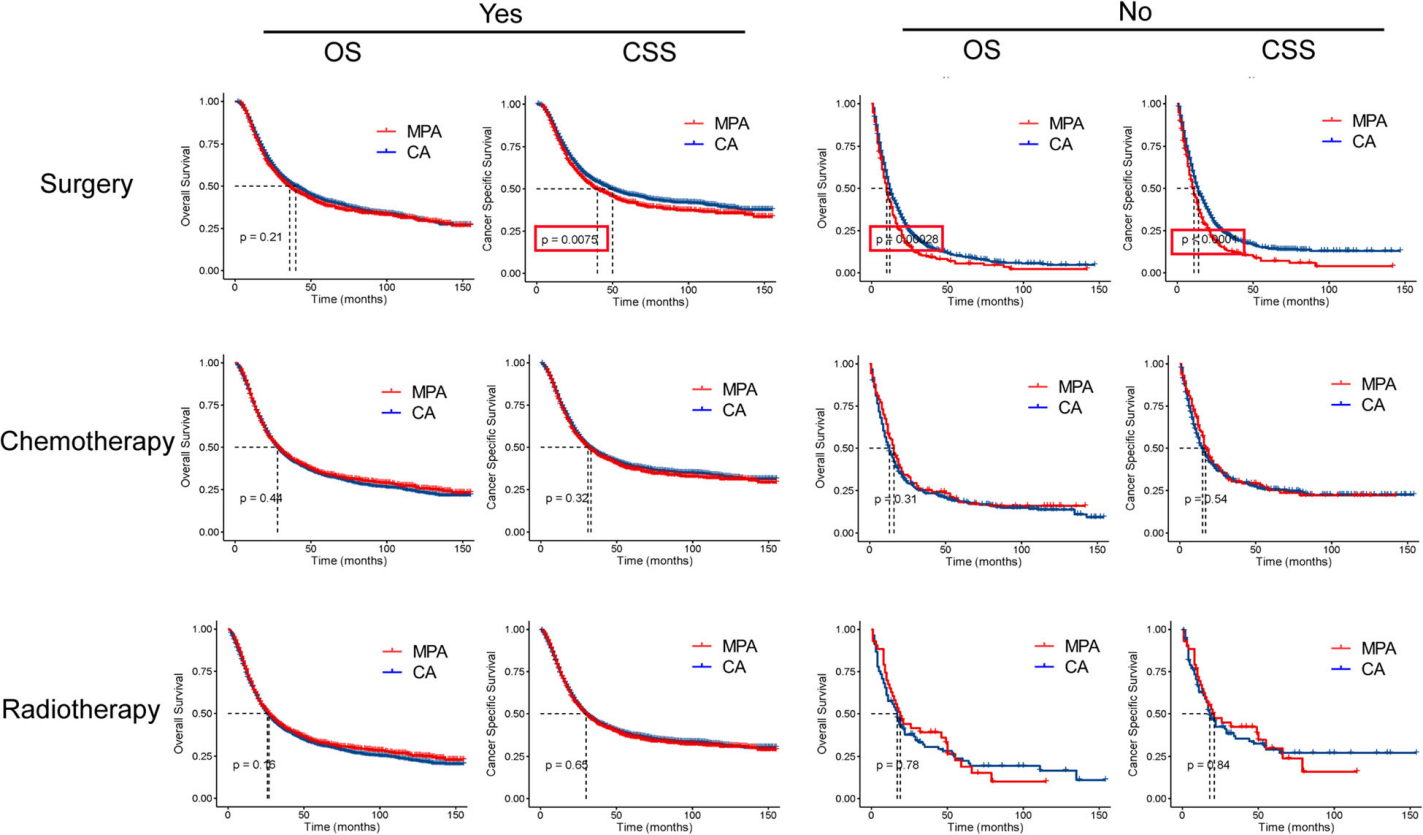
Overall survival (OS) and cancer specific survival (CSS) analysis by mucin-producing adenocarcinoma (MPA) and conventional adenocarcinoma (CA) in different therapeutic groups of gastric cancer patients..

To further determine whether MPA patients show similar survival time with CA patient in different therapeutic subgroups, we compared the survival curves. As shown in Fig. 7, MPA patients had shorter CSS time than CA patients after surgery, but there was no difference in OS time between the two groups. MPA patients without surgery had shorter OS and CSS time than that in CA patients. MPA patients had similar OS and CSS time with CA patients with or without radiotherapy/ chemotherapy.

### Survival analysis following treatments

**Fig. 8:**
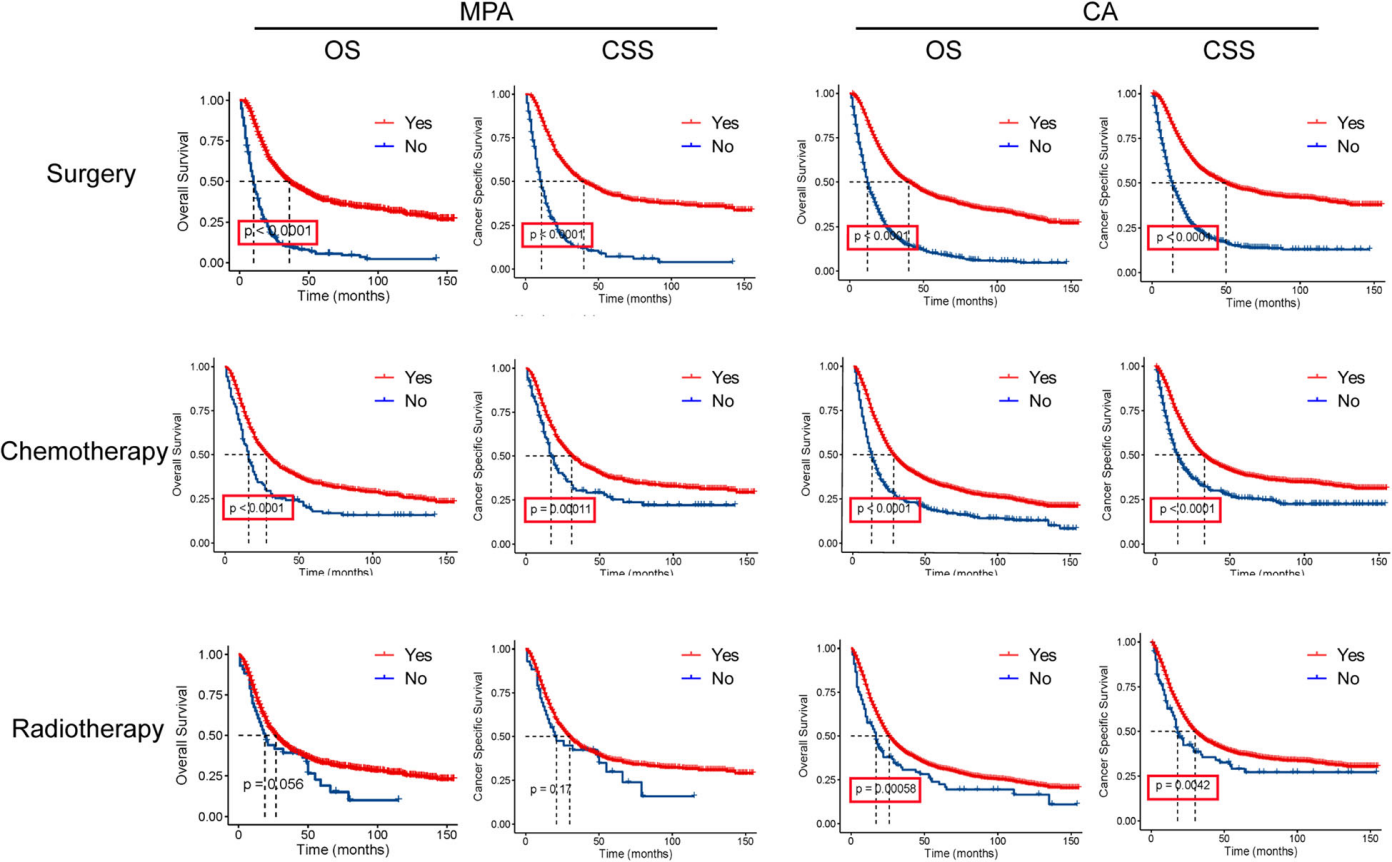
Overall survival (OS) and cancer specific survival (CSS) analysis by clinical therapies in mucin-producing adenocarcinoma (MPA) and conventional adenocarcinoma (CA) gastric cancer patients

To investigate whether MPA patients obtained a survival benefit from different clinical therapies, we compared the OS and CSS time periods. As shown in Fig. 8, MPA patients who were treated with surgery exhibited longer OS and CSS time than those without surgery (both *P* < 0.05). MPA patients treated with chemotherapy exhibited longer OS and CSS time than those without chemotherapy (both P < 0.05). MPA patients treated with radiotherapy exhibited similar OS and CSS time with those without radiotherapy (both *P* > 0.05). Patients with CA obtained longer OS and CSS time when treated with surgery, chemotherapy and radiotherapy (All *p* < 0.05).

### Adjusted survival analysis

Cox regression was applied to analyze the association between histological type and overall survival following adjustment for baseline differences. As shown in Table 2, patients with MPA did not exhibit a significantly different hazard of death following adjustment for these variables (*P* = 0.15). For both groups, the significant independent prognostic factors were age, race, tumor differentiation, clinical stage, surgery and chemotherapy (All *P* < 0.05). MPA histology was not a prognostic factors.

**Table 2:** Univariate and multivariate analysis of overall survival for gastric cancer patients

	Univariate analysis				Multivariate analysis			
	**HR**	**95% CI**		**p**	**HR**	**95% CI**		**p**
		**Lower**	**Upper**			**Lower**	**Upper**	
**MPA vs.CA**	0.94	0.88	1.02	0.15	–	–	–	–
**Age**	1.48	1.38	1.59	0.00	1.22	1.14	1.31	0.00
**Race**	0.84	0.8	0.88	0.00	0.9	0.86	0.94	0.00
**Gender**	1.08	1	1.16	0.03	1.04	0.97	1.12	0.24
**Differentiation**	1.07	1	1.15	0.03	1.22	1.14	1.31	0.00
**Clinical stage**	1.1	1.06	1.15	0.00	1.31	1.23	1.39	0.00
**T stage**	1.08	1.03	1.13	0.00	0.98	0.92	1.04	0.59
**N stage**	1.03	0.98	1.08	0.22	–	–	–	–
**Surgery**	0.32	0.3	0.35	0.00	0.31	0.29	0.34	0.00
**Chemotherapy**	0.55	0.5	0.61	0.00	0.69	0.62	0.76	0.00
**Radiation**	0.69	0.58	0.83	0.00	0.88	0.72	1.08	0.23

Notes: HR, hazard ratio. CI, confidence intervals. MPA. mucin-producing adenocarcinoma. CA, conventional adenocarcinoma

## Discussion

MPA involves tumors, such as mucinous adenocarcinoma and signet ring cell carcinoma and produces mucin, which is observed by histological analysis [4]. Both of these two histological types are defined as undifferentiated [3, 25]. Generally, the degree of cancer cell differentiation is associated with cancer aggressiveness. Undifferentiated cancer correlates with aggressive growth, invasion, metastasis and poor prognosis [3, 26].

However, the prognosis of gastric cancer patients with MPA is still controversial and unclear. Several studies have reported that MPA correlates with poor prognosis compared with other histological types [7–10], while other reports have not yielded significant differences in disease prognosis [11–15]. Moreover, it has also been shown that signet ring cell carcinoma is associated with a favorable prognosis than other types of gastric cancer [19–22].

Therefore, the present study retrospectively analyzed 1515 MPA gastric cancer patients from the SEER database in order to assess the prognostic value of MPA. In the present study, the proportion of MPA in gastric cancer was approximately 18.1% (14,243/78303), which was considerably higher than that noted in previous studies (2.6–6.6%) [3, 12, 14]. The proportion of CA in gastric cancer was approximately 46.7% (36,602/78303). This evidence suggested that MPA was a rather rare histological type of gastric cancer. The data indicated that patients in the MPA group were younger, more females, more black people, more poor/undifferentiated tumors, later clinical stage, more T3, T4, N2 stages, more surgery and chemotherapy than that of the CA group, which suggested that MPA was an aggressive histological type. No significant differences were noted in the OS and CSS time periods between the MPA and the CA groups, suggesting that gastric cancer patients with MPA had similar prognosis with those with CA. However, for patients with poor/ undifferentiated tumor, clinical stage I, T1 and T2, MPA patients had longer OS time than CA patients. MPA patients had shorter CSS time than CA patients after surgery, but there was no difference in OS time between the two groups. MPA patients without surgery had shorter OS and CSS time than that in CA patients. These results suggested that MPA patients had similar survival time with CA patients on the whole, but MPA was an aggressive histological type in some way. MPA was not a prognostic factor of survival. The results were basically in accordance with those from Zheng et al., who compared the CSS of esophageal cancer patients with MPA or CA and found similar survival time periods between the two groups [27].

With regard to the treatment of MPA, the patients who were treated with surgery or chemotherapy had longer OS and CSS time periods than those without treatment, suggesting that surgery and chemotherapy were effective treatments for early and localized gastric cancer patients with MPA. However, MPA patients treated with radiotherapy exhibited similar OS and CSS time with those without radiotherapy in this study, which indicated that radiotherapy may not be an effective treatment for gastric cancer patients with MPA. However, pertinent information regarding radiotherapy was not available in the SEER database, which could have affected the results. Therefore, further investigations are required to identify the practical and clinical significance of radiotherapy for patients with MPA. Moreover, the therapeutic roles of chemotherapy and radiotherapy for patients with metastatic MPA were not conducted in this study and require further studies.

The present study has its limitations. Firstly, it was a retrospective study and the baseline characteristics of both groups were different, which unavoidably contained selection bias. Secondly, due to the rarity of MPA cases and the exclusion of MPA patients with missing data, the number of patients with MPA used in the present study was low, compared with CA, which may affect the results in a way. Thirdly, the SEER database does not collect several important information, such as the type and extent of surgery, details on chemotherapy and radiotherapy and disease recurrence, which could have affected the results. Therefore, larger studies are required to further determine the clinical and pathological roles of MPA.

## Conclusions

MPA is a rather rare histological type of gastric cancer. Patients with MPA had similar prognosis with those with CA. MPA was not an prognostic factor of survival. Surgery and chemotherapy were effective treatments for gastric cancer patients with MPA.

## Data Availability

The data used and analyzed during the study are available in SEER database (http://seer.cancer.gov).
